# NLRP3/Caspase-1-Mediated Pyroptosis Drives a Brain–Lesion Neuroimmune Axis in Endometriosis-Associated Pain: Molecular Mechanisms and Transcranial Direct Current Stimulation Intervention

**DOI:** 10.3390/biomedicines14081713

**Published:** 2026-07-30

**Authors:** Ping Zheng, Aihong You, Yong Fan

**Affiliations:** 1 Department of Obstetrics and Gynecology, Beijing Chao-Yang Hospital, Capital Medical University, Beijing 100020, China; 2 Department of Obstetrics and Gynecology, The Third Affiliated Hospital of Guangzhou Medical University, Guangzhou 510150, China

**Keywords:** endometriosis, pain, pyroptosis, NLRP3 inflammasome, neuroimmune modulation, transcranial direct current stimulation

## Abstract

**Background/Objectives**: The NLRP3 inflammasome–Caspase-1–IL-1β pyroptotic axis participates in peripheral inflammatory responses, yet its function in peripheral–central neuroimmune crosstalk underlying endometriosis (EM)-associated pain remains unclear. This study aimed to clarify whether NLRP3-mediated pyroptosis establishes a brain–lesion neuroimmune axis connecting ectopic lesion inflammation with central neuroimmune remodeling and to explore the therapeutic mechanism of transcranial direct current stimulation (tDCS). **Methods**: An EM rat model was established to detect NLRP3 pathway expression in ectopic lesions and anterior cingulate cortex (ACC), together with central nervous system pathological alterations. Animals received tDCS intervention to evaluate inflammatory, neuropathological and pain behavioral changes. The closed-loop brain–lesion regulatory circuit was further interpreted. In a clinical cohort including 40 EM patients, pain and quality-of-life scores were compared between active and sham tDCS groups. **Results**: NLRP3, Caspase-1 and IL-1β were upregulated in ectopic lesions and ACC of EM rats, accompanied by ACC mitochondrial injury, microglial activation and thalamic demyelination. tDCS inhibited pyroptosis-related molecules, decreased systemic proinflammatory cytokines, improved central pathological lesions and relieved pain hypersensitivity. Mechanically, top-down descending pain inhibitory pathways, vagal cholinergic anti-inflammatory pathway and the HPA axis jointly mediate therapeutic effects, whereas circulating cytokines and visceral afferents transmit peripheral inflammatory signals to the brain. Clinical data demonstrated that active tDCS effectively alleviated EM-related pain and improved patients’ quality of life. **Conclusions**: NLRP3-mediated pyroptosis acts as a key mediator linking peripheral and central neuroimmune communication. Targeting this pathway via tDCS interrupts the inflammation–pain vicious cycle through multiple neuroregulatory pathways and remodels the central neuroimmune microenvironment in endometriosis.

## 1. Introduction

Endometriosis (EM) is a highly prevalent chronic condition affecting women of reproductive age, with a global prevalence reaching up to 10%—approximately 190 million women worldwide [1,2,3]. The hallmark clinical manifestation is pain; approximately 70% of patients experience chronic pelvic pain, of whom 30% develop refractory severe pain (visual analog scale [VAS] ≥ 5), characterized by persistent dysmenorrhea, deep dyspareunia, and non-menstrual lower abdominal discomfort [4,5]. These symptoms profoundly compromise fertility, mental well-being, and social functioning. Current mainstream therapeutic strategies face considerable limitations: pharmacologically, nonsteroidal anti-inflammatory drugs (NSAIDs) provide only transient symptomatic relief and carry a substantial risk of gastrointestinal injury with prolonged use. Hormonal treatments, such as gonadotropin-releasing hormone agonists (GnRH-a), can suppress ectopic lesion growth but are associated with adverse effects including bone loss and vasomotor symptoms, with pain recurrence rates exceeding 40% within six months after discontinuation [6,7,8]. Surgical intervention, while capable of directly excising lesions, is associated with a 30–50% recurrence rate within five years, and repeated procedures may further diminish ovarian reserve [9]. The fundamental shortcoming of these conventional approaches lies in their failure to effectively disrupt the “pain–inflammation” vicious cycle. Consequently, there is a pressing need to investigate novel noninvasive strategies targeting neuroimmune pathways.

Emerging research indicates that the core mechanism underlying endometriosis-associated pain is intricately linked to the neuroimmune cascade mediated by the NLRP3 inflammasome. Peripherally, damage-associated molecular patterns (DAMPs) released from ectopic endometrial tissues persistently activate the NLRP3 inflammasome in local macrophages and endometrial stromal cells [10,11,12]. Upon assembly, the inflammasome recruits and activates caspase-1 via the adaptor protein ASC, leading to the maturation and secretion of pro-inflammatory cytokines interleukin-1β (IL-1β) and IL-18 [13,14,15]. These cytokines directly stimulate pelvic nerve terminals, inducing peripheral sensitization. Clinical studies have confirmed that serum IL-1β levels in patients with severe EM pain are elevated 2.5-fold compared with healthy controls and show a significant positive correlation with VAS scores [16]. Centrally, peripheral nociceptive signals are transmitted via afferent nerves to the brain’s pain-processing network, activating microglia in the anterior cingulate cortex (ACC) and periaqueductal gray (PAG), which triggers NLRP3 inflammasome assembly within the brain. Microglial-derived IL-1β enhances glutamatergic synaptic transmission, promoting central sensitization characterized by reduced pain thresholds and heightened pain sensitivity [17]. Animal experiments have clearly demonstrated that in EM model rats, ACC NLRP3 expression is increased 3.2-fold relative to controls and strongly inversely correlates with shortened latency in the hot plate test. This “peripheral sensitization–central amplification” bidirectional pathway constitutes the pathological foundation of chronic pain in endometriosis.

Transcranial direct current stimulation (tDCS), an emerging noninvasive neuromodulation technique, offers a novel approach to addressing these therapeutic challenges [18,19]. By applying a weak direct current (typically 1–2 mA) through scalp electrodes [20], anodal stimulation can enhance the excitability of the dorsolateral prefrontal cortex (DLPFC) and activate descending pain inhibitory pathways (e.g., the PAG–RVM–spinal cord dorsal horn pathway), thereby modulating pain information integration [21]. Compared with conventional therapies, tDCS offers several distinct advantages.

## 2. Materials and Methods

### 2.1. Study Design

This study aimed to comprehensively evaluate the efficacy of transcranial direct current stimulation (tDCS) in the management of endometriosis (EM)-related pain and to explore its potential underlying mechanisms. The research comprised two interrelated components—a clinical study and an animal experiment—designed to complement each other and provide comprehensive translational evidence.

### 2.2. Clinical Study

#### 2.2.1. Participants

All participants were recruited between January 2025 and June 2025 from the gynecology outpatient clinic at Beijing Chao-Yang Hospital, Capital Medical University. All subjects provided written informed consent prior to completing the questionnaires and receiving tDCS intervention.

The inclusion criteria were as follows: female patients aged 18–45 years; confirmed diagnosis of endometriosis via imaging examinations (e.g., transvaginal ultrasound, magnetic resonance imaging) or surgical pathology; no recent use (within 1 month) of steroid hormones or analgesic medications; and the presence of chronic pelvic pain with a Visual Analog Scale (VAS) score ≥ 5.

The exclusion criteria included concurrent diagnosis of other chronic pain disorders (e.g., interstitial cystitis, chronic pelvic inflammatory disease), history of neurological or psychiatric diseases (e.g., epilepsy, depression, schizophrenia), ongoing pain management interventions (e.g., acupuncture, nerve block), presence of intracranial metal implants, and pregnancy or lactation status.

A prospective sample size estimation was performed for this exploratory clinical study using GraphPad Prism 10.0 software, based on the primary outcome of the Endometriosis Pain Scale (EPS) score. A clinically significant between-group difference in EPS score was defined as 2.0 points, with an assumed standard deviation (SD) of 1.5 points derived from prior studies of neuromodulation for chronic pelvic pain. A two-sided type I error (α) of 0.05 and a statistical power (1−β) of 80% were adopted as the calculation criteria. The minimum required sample size per group was calculated to be 18 participants. To account for potential data incompleteness and ensure robust analysis, we enrolled 20 participants per group (total n = 40), providing adequate statistical power for the between-group comparison of the primary outcome. The baseline clinical characteristics of the two groups were comparable (*p* > 0.05), ensuring the reliability of subsequent efficacy comparisons.

#### 2.2.2. Intervention

tDCS equipment: A transcranial direct current stimulator (model: MBM-I, batch number: 020090018), supplied by Jiangxi Huaheng Jingxing Medical Technology Co., Ltd. (Nanchang, China), was employed for all clinical interventions. This equipment was of the same model as that used in the animal experiments, with stimulation parameters adjusted for human subjects.

tDCS stimulation: All patients received a standard 20 min tDCS stimulation protocol. The anode electrode was placed at the F3 position (according to the international 10–20 system), and the cathode electrode was positioned at the Fp2 position on the contralateral supraorbital region. The current intensity was set at 2 mA, and stimulation was administered once daily for 10 consecutive days. The stimulation procedure was closely monitored to ensure patient comfort and safety.

Sham stimulation: Patients in the sham stimulation group received the same electrode placement as the tDCS treatment group to eliminate the placebo effect of electrode positioning. However, the current intensity was set to 0 mA throughout the 20 min session, which was conducted once daily for 10 consecutive days, consistent with the treatment frequency of the tDCS group.

#### 2.2.3. Evaluation Indicators: Pain Assessment

**Figure 1:**
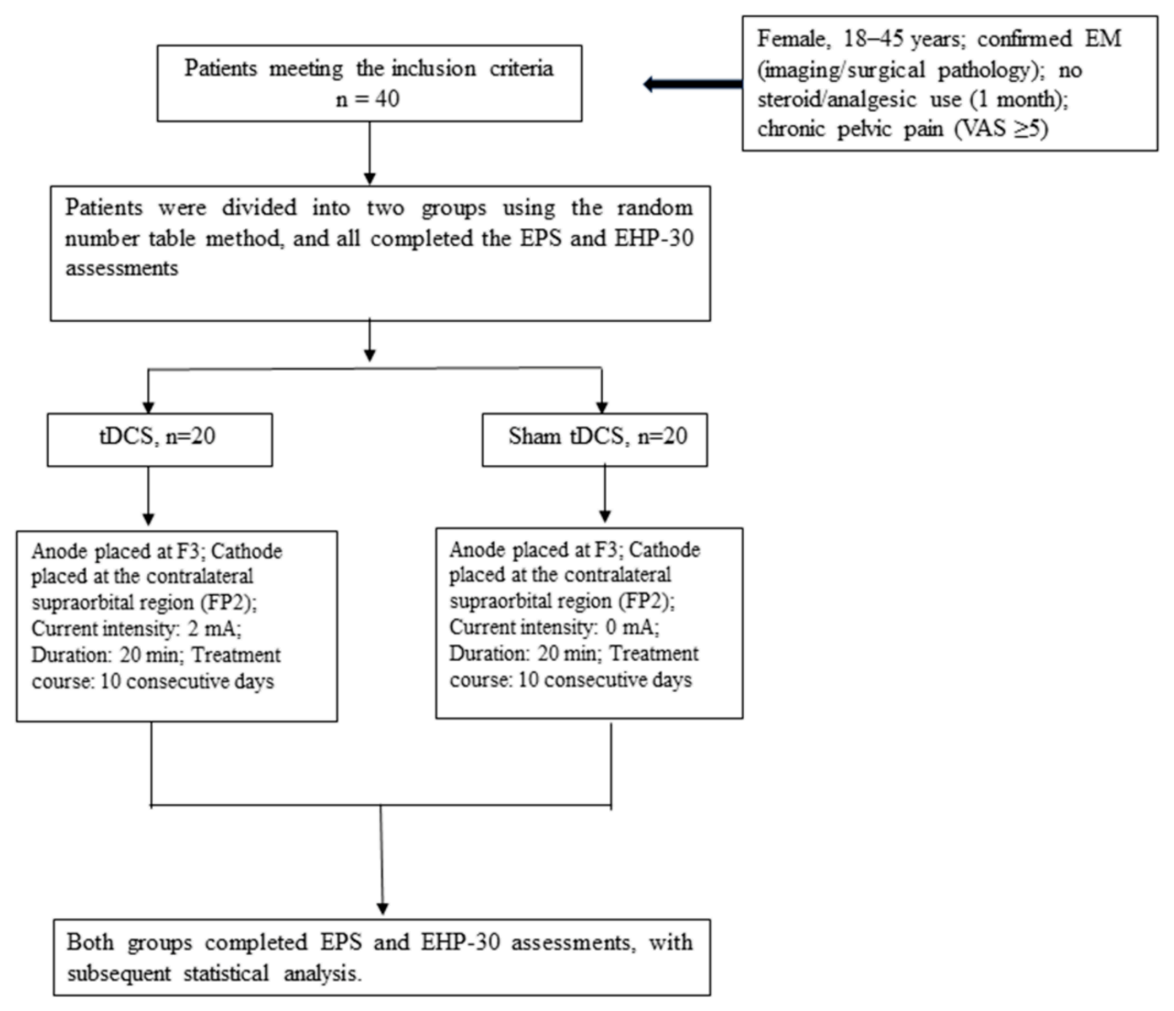
Patient tDCS Treatment and Assessment Flow. Flow diagram illustrating the tDCS treatment administration and outcome assessment protocol for endometriosis (EM) patients.

Pain-related outcomes were assessed at three time points: before treatment (baseline), 1 day after completion of treatment, and 10 days after completion of treatment. The Endometriosis Pain Scale (EPS) was used to specifically evaluate the severity of EM-related pain (e.g., dysmenorrhea, dyspareunia, chronic pelvic pain), while the Endometriosis Health Profile-30 (EHP-30) was adopted to assess the impact of EM-related pain on patients’ quality of life. All assessments were performed by trained investigators blinded to group allocation to minimize subjective bias. The design and workflow of the clinical study are illustrated in Figure 1.

### 2.3. Animal Experiments

#### 2.3.1. Model Establishment

The animal experiment was conducted from July 2025 to December 2025 at the Experimental Animal Center of Beijing Chao-Yang Hospital, Capital Medical University. All rat experiments were performed in accordance with ARRIVE guidelines under approved animal ethical protocols.

Endometriosis (EM) was induced in female Sprague–Dawley (SD) rats (220 ± 20 g, 8–10 weeks old) via autologous uterine tissue transplantation, as previously described. All rats were specific pathogen-free (SPF) grade and were purchased from the Experimental Animal Center of Capital Medical University [license No. SCXK (Beijing) 2024-0001] to ensure consistent health status. Successful establishment of the EM model was confirmed four weeks postoperatively based on the following criteria: ectopic lesion diameter > 3 mm and hot plate latency shortened by ≥30% compared with sham-operated rats. The rats were then randomly divided into two groups using a random number table: EM pain rats (n = 28) were allocated to either the tDCS group (n = 14) or the sham stimulation group (n = 14).

#### 2.3.2. Intervention

tDCS equipment (for animal experiments): Transcranial DC Stimulator, model: MBM-I, batch number: 020090018, was provided by Jiangxi Huaheng Jingxing Medical Technology Co., Ltd. (Nanchang, China).

Anesthesia (in accordance with American Veterinary Medical Association [AVMA] guidelines): All interventions were performed under 1% pentobarbital sodium anesthesia (50 mg/kg, intraperitoneal injection, sterile preparation). The anesthesia depth was monitored by assessing the disappearance of the tail-flick reflex and corneal reflex prior to stimulation; supplementary anesthesia (10% of the initial dose) was administered if spontaneous movement occurred during the intervention to minimize animal discomfort and ensure stable positioning. Following stimulation, rats were placed in a warm cage (37 ± 1 °C) for recovery until the restoration of normal locomotor and feeding behavior.

tDCS group: The anode was positioned on the forehead (anteroposterior [AP]: +2.0 mm, mediolateral [ML]: 0 mm, relative to bregma), while the cathode was placed on the ipsilateral shoulder. A constant current of 1 mA was applied for 20 min per day over a continuous period of 10 days, with stimulation parameters referenced to the rat brain stereotaxic atlas [22].

Sham stimulation group: Under the same anesthesia conditions, the anode and cathode were placed at identical positions as in the tDCS group. However, no electrical current was delivered during the 20 min sessions conducted over 10 days, to eliminate the confounding effects of electrode placement and anesthesia.

#### 2.3.3. Euthanasia Method

After completion of all experimental assessments, the rats were euthanized by an overdose of pentobarbital sodium (150 mg/kg, intraperitoneal injection). Euthanasia efficacy was confirmed by the permanent loss of the corneal reflex, cessation of spontaneous breathing, and the absence of a detectable heart rate (monitored by palpation of the abdominal aorta) for more than 5 min. Tissue collection was performed immediately after confirmation of euthanasia to ensure tissue integrity.

#### 2.3.4. Assessment Parameters

All assessments were performed by investigators blinded to group allocation to minimize subjective bias.

Behavioral assessment: Hind limb withdrawal latency was measured using the hot plate test (55 °C, maintained at a constant temperature). Each rat was placed on the hot plate, and the latency to hind limb withdrawal (licking or lifting of the hind limb) was recorded, with a cut-off time of 60 s to prevent tissue damage. Each measurement was repeated three times at 5 min intervals, and the average value was calculated for statistical analysis.

Molecular analysis: Immunohistochemistry (IHC) was used to quantify Caspase-1 and IL-1β expression in lesions and the ACC. Paraffin-embedded sections were dewaxed, rehydrated, and subjected to antigen retrieval (EDTA buffer, pH 9.0), followed by primary and secondary antibody incubation. Positive expression was visualized by 3,3′-diaminobenzidine (DAB) staining, images were captured under a light microscope, and quantitative analysis was performed using Image-Pro Plus 6.0 (Media Cybernetics, Rockville, MD, USA) with the average optical density (AOD) as the measurement indicator.

Transmission electron microscopy (TEM) analysis: TEM analyses were performed on eutopic endometrium, ectopic lesions, and three brain regions (cingulate cortex, hippocampus, and thalamus) from each rat in both groups, following the protocol described by Ding et al. Tissue sections were fixed in electron microscope fixative for 2–4 h and then in 1% osmium tetroxide (0.1 M phosphate buffer, pH 7.4, 20 °C) for 2 h, dehydrated in graded ethanol, and embedded in epoxy resin. Ultrathin sections (0.1 μm) were stained with uranyl acetate and lead citrate, observed under a TEM (HT7700, HITACHI, Tokyo, Japan), and representative images were captured.

### 2.4. Statistical Analysis

Data analysis was performed using GraphPad Prism 10.0 software (GraphPad Software, Inc., La Jolla, CA, USA) and SPSS 26.0 (IBM Corp., Armonk, NY, USA). All data were expressed as the mean ± standard deviation ($\bar{x}$ ± s). Normality was assessed using the Shapiro–Wilk test, and homogeneity of variance was verified using Levene’s test. For clinical EPS and EHP-30 scores and rat hot plate withdrawal latency, within-group changes across the three assessment time points were evaluated by one-way ANOVA, and between-group comparisons at each time point were performed using independent samples *t*-tests. For immunohistochemical optical density (Caspase-1, IL-1β AOD) and serum biomarkers (IL-1β, TNF-α, LDH), independent samples *t*-tests were used. A *p* value < 0.05 was considered statistically significant. We acknowledge that, because the clinical cohort was diagnosed by imaging (transvaginal ultrasound and MRI) without systematic laparoscopic r-AFS surgical staging and most participants had not undergone surgery, repeated-measures ANOVA and linear mixed-effects models were not performed in the present study; the statistical approach is therefore noted as a limitation.

### 2.5. Main Materials and Reagents

ELISA kits: Rat IL-1β (Cat. No.: E-EL-R0012c, batch No.: 20250315) and TNF-α (Cat. No.: E-EL-R0019c, batch No.: 20250316), Wuhan Elabscience Biotechnology Co., Ltd. (Wuhan, China).

Immunohistochemical reagents: Caspase-1 (Cat. No.: bs-20714R, batch No.: 20250208, Bioss, Beijing, China); IBA-1 (Cat. No.: ab178846, batch No.: GR3578885-2, Abcam, Cambridge, UK); IL-1β (Cat. No.: PB0446, batch No.: 250203, Boster, Wuhan, China); TNF-α (Cat. No.: PT516, Beyotime, Shanghai, China); LDH (Cat. No.: BC03246, EK-Bio, Shanghai, China); DAB kit (Cat. No.: ZLI-9018, batch No.: 20250302, ZSGB-BIO, Beijing, China); EDTA buffer (pH 9.0, Cat. No.: C1032, batch No.: 20250210, Beyotime, China).

## 3. Results

### 3.1. Clinical Study Results

#### 3.1.1. Baseline Characteristics of Participants

Forty eligible female patients with endometriosis (EM)-related chronic pelvic pain were enrolled, all of whom completed the 10-day intervention and follow-up without dropout. Participants were randomly assigned to either the tDCS treatment group or the sham stimulation control group (20 patients each) using a random number table. The baseline characteristics were comparable between the two groups (all *p* > 0.05): the tDCS group had VAS 8.00 ± 1.60, age 37.00 ± 2.10 years, EPS 7.25 ± 1.14, and EHP-30 74.92 ± 6.78; the sham group had VAS 8.00 ± 1.40, age 38.00 ± 2.30 years, EPS 7.30 ± 1.21, and EHP-30 74.56 ± 6.69. Independent samples *t*-tests confirmed good baseline comparability (t = 0.42, *p* = 0.68 for VAS; t = 1.58, *p* = 0.12 for age), supporting the validity of the subsequent efficacy comparisons.

#### 3.1.2. Effects of tDCS on EPS and EHP-30 Scores

Pain severity (EPS) and quality of life (EHP-30) were evaluated at baseline, 1 day post-treatment, and 10 days post-treatment by investigators blinded to group allocation. After 10 days of intervention, the two groups exhibited distinct patterns of score changes.

In the tDCS group, the EPS scores decreased significantly from the baseline (7.25 ± 1.14) to 4.75 ± 0.85 (1 day post-treatment) and 3.65 ± 0.67 (10 days post-treatment) (one-way ANOVA, all *p* < 0.001). The EHP-30 scores also declined significantly from 74.92 ± 6.78 (baseline) to 51.85 ± 5.87 (1 day) and 39.12 ± 5.09 (10 days) (all *p* < 0.001), indicating that tDCS effectively relieved pain and improved the quality of life.

In contrast, the sham group showed no significant changes in the EPS or EHP-30 scores (all *p* > 0.05): the EPS was 7.30 ± 1.21 (baseline), 6.85 ± 1.32 (1 day), and 7.05 ± 1.28 (10 days); the EHP-30 was 74.56 ± 6.69 (baseline), 72.98 ± 6.51 (1 day), and 72.65 ± 6.40 (10 days).

Intergroup comparison at 10 days post-treatment revealed significantly lower EPS (t = 9.82, *p* < 0.001) and EHP-30 (t = 10.56, *p* < 0.001) scores in the tDCS group (detailed data in Table 1).

### 3.2. Animal Experiment Results

#### 3.2.1. Success of Endometriosis Model Establishment and tDCS Intervention

**Figure 2:**
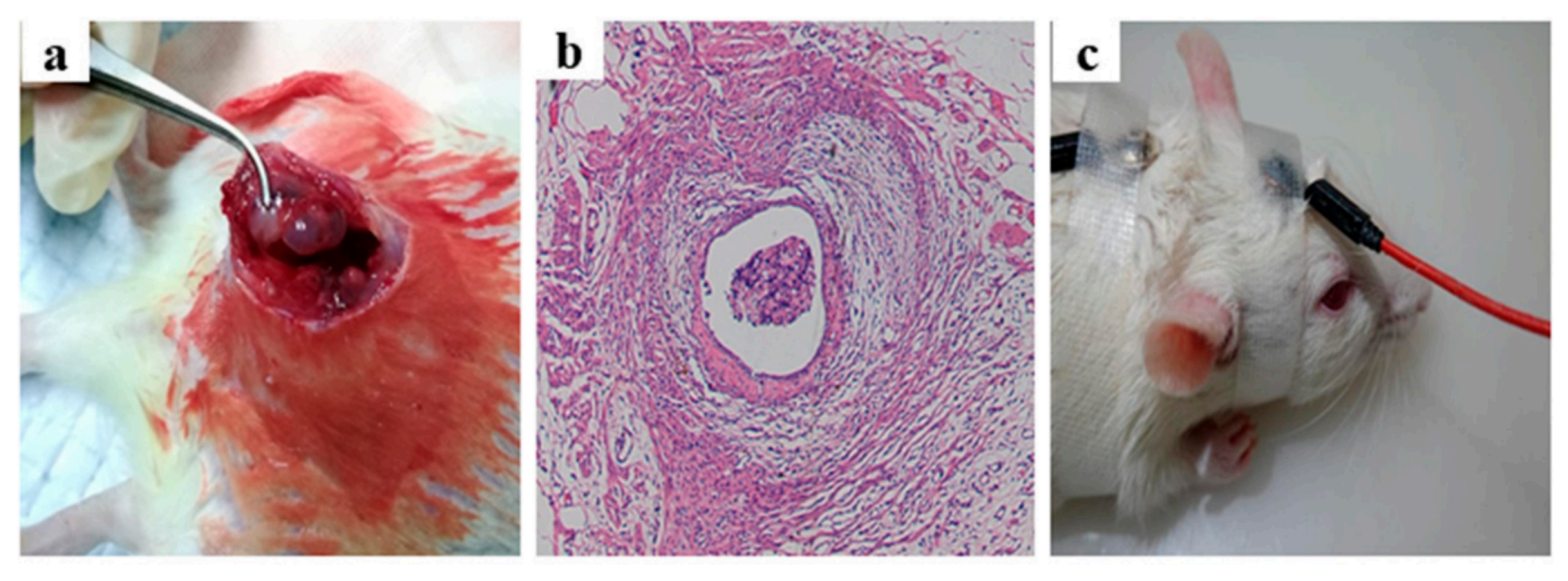
EM Rat Model Establishment and tDCS Intervention. (**a**) Abdominal EM rat model established by autologous uterine tissue transplantation; (**b**) HE staining (×200) showing endometriotic glandular structures in abdominal cyst tissue; (**c**) tDCS intervention in rats, illustrating the placement positions of the anode and cathode electrodes.

Specific pathogen-free (SPF) female Sprague–Dawley (SD) rats (220 ± 20 g, 8–10 weeks old) were used to establish EM models via autologous uterine tissue transplantation, as previously described. Model success was confirmed four weeks postoperatively by the presence of abdominal ectopic lesions with a diameter >3 mm and a ≥30% reduction in hot plate latency compared with the sham-operated rats. The rats were then randomly assigned to two groups using a random number table: EM pain rats (n = 28) were allocated to either the tDCS group (n = 14) or the sham stimulation group (n = 14), as shown in Figure 2.

#### 3.2.2. Effects of tDCS on Pain Threshold in EM Rats (Hot Plate Test)

The hot plate test (55 °C, 60 s cut-off) was employed to evaluate pain thresholds in EM rats, using hind limb withdrawal latency as the primary index. Each rat was measured three times at 5 min intervals, and the average value was used for statistical analysis.

At baseline, no significant difference in withdrawal latency was observed between the two groups (sham: 10.86 ± 0.71 s; tDCS: 11.32 ± 0.78 s; t = 0.814, *p* = 0.4229), indicating comparable baseline pain status.

**Figure 3:**
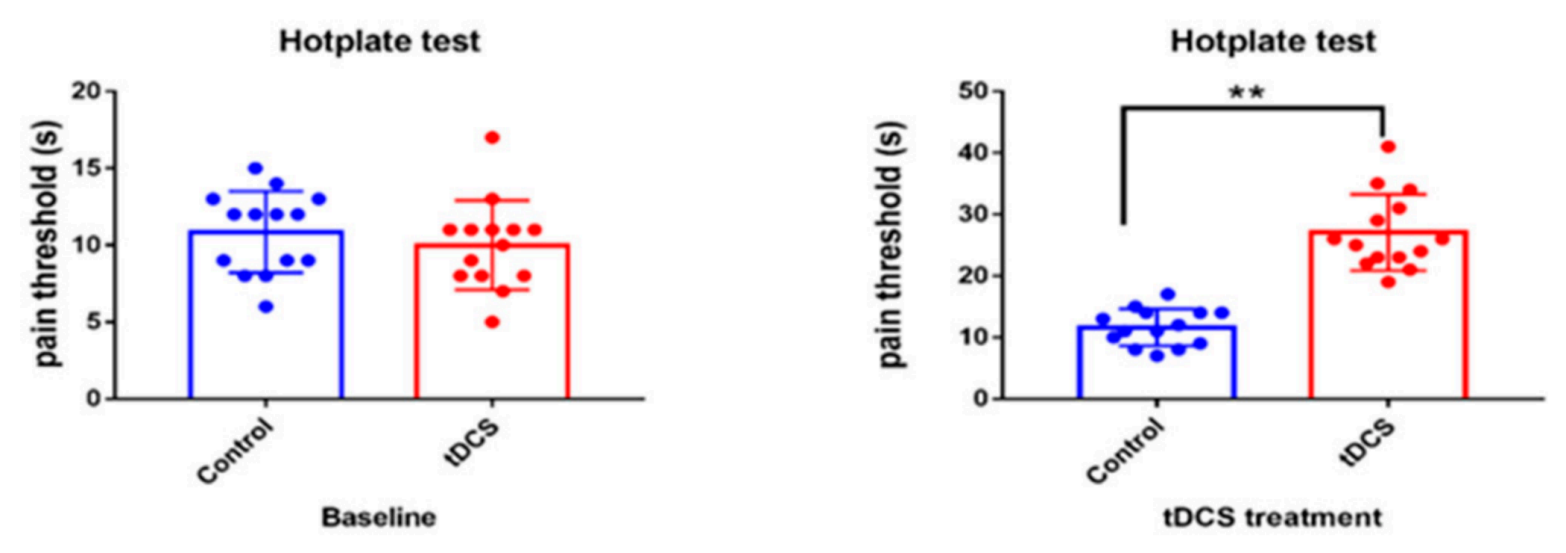
Comparison of pain thresholds before and after treatment in rats of two EM pain groups. Data are expressed as $\bar{x}$ ± s (n = 14 per group). No significant difference in hind limb withdrawal latency was observed between the two groups at baseline (*p* > 0.05). After tDCS treatment, latency was significantly longer in the tDCS group than in the sham group (** *p* < 0.001). Hot plate temperature: 55 °C; cut-off time: 60 s.

After 10 consecutive days of intervention (1 mA, 20 min/day), the withdrawal latency in the tDCS group increased significantly to 27.07 ± 1.66 s (vs. baseline, *p* < 0.001), whereas the sham group showed no significant change (11.64 ± 0.80 s, *p* > 0.05). Intergroup comparison revealed a significant difference (t = 26.47, *p* < 0.001), demonstrating that tDCS effectively elevated the pain thresholds in EM rats, as shown in Figure 3.

### 3.3. Effects of tDCS on Eutopic and Ectopic Endometrial Lesions and Brain Region Morphology in EM Rats

Following the intervention, rats were euthanized by intraperitoneal overdose of 1% pentobarbital sodium (50 mg/kg), and tissues from the anterior cingulate cortex (ACC), hippocampal CA1 region, and thalamus were immediately collected for hematoxylin and eosin (HE) staining and transmission electron microscopy (TEM) to evaluate the morphological and ultrastructural changes.

#### 3.3.1. Eutopic and Ectopic Endometrial Lesions

**Figure 4:**
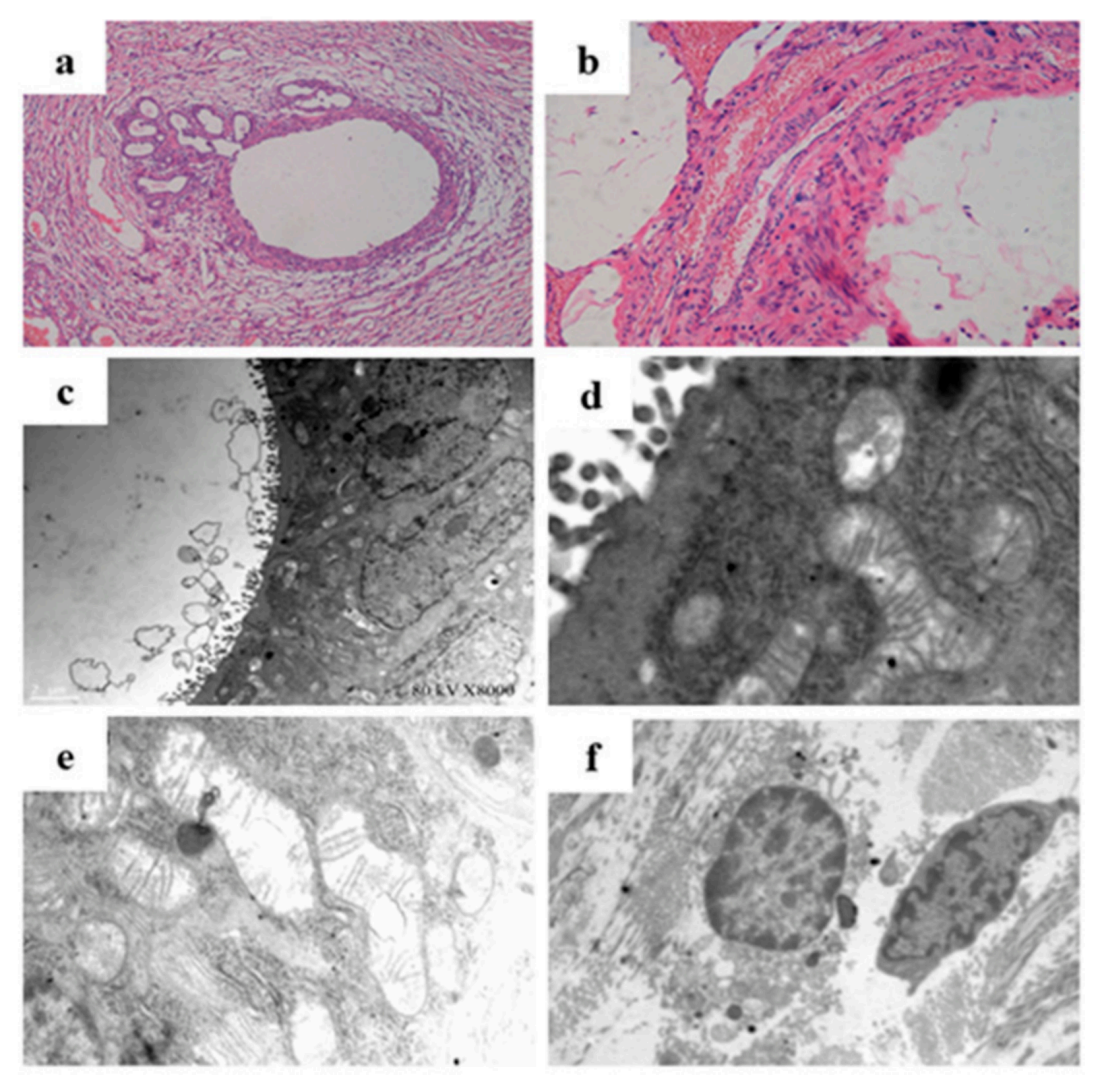
Morphological changes in the eutopic endometrium and ectopic lesions. (**a**) Normal rat endometrium (HE staining, ×40); (**b**) endometriotic lesions in rat model (HE staining, ×200); (**c**) normal rat endometrium (TEM, ×8000). Transmission electron microscopy of eutopic endometrium and ectopic lesions showed (**d**) mitochondria in the eutopic endometrium with intact structure and tightly arranged cristae (TEM, ×30,000); (**e**) mitochondria in the ectopic lesions undergoing fission, exhibiting shrinkage, rounding, loose arrangement, and disruption of cristae (TEM, ×30,000); (**f**) cells in the ectopic lesions exhibiting apoptotic morphology (TEM, ×30,000).

HE staining and transmission electron microscopy revealed marked morphological and ultrastructural differences between the eutopic endometrium and ectopic lesions. As shown in Figure 4, the eutopic endometrium displayed a normal histological architecture under HE staining (a), whereas typical endometriotic glandular structures were observed in the ectopic lesions (b). TEM examination further demonstrated that mitochondria in the eutopic endometrium exhibited intact structures with tightly arranged cristae (d), while mitochondria in the ectopic lesions underwent fission, characterized by shrinkage, rounding, loosened cristae arrangement, and even cristae disruption (e). Additionally, cells in the ectopic lesions exhibited apoptotic morphology (f). These findings indicate that ectopic endometrial tissue undergoes significant ultrastructural damage, including mitochondrial dysfunction, which may serve as an upstream trigger for NLRP3 inflammasome activation.

#### 3.3.2. Anterior Cingulate Cortex (ACC)

**Figure 5:**
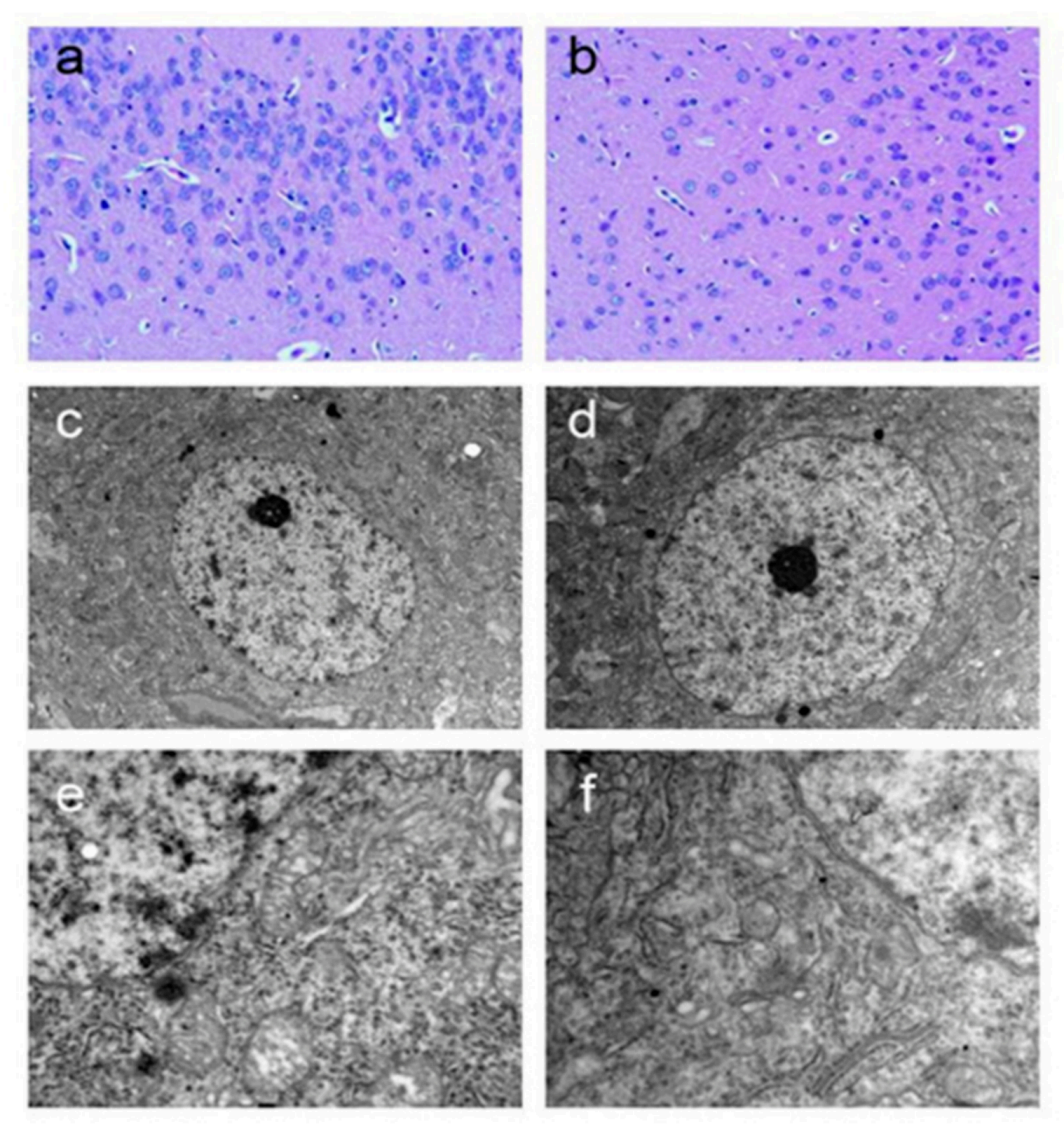
Morphological changes in ACC in EM rats (HE staining and TEM). HE staining (×200) and TEM ((**c**,**d**) ×10,000; (**e**,**f**) ×30,000). (**a**,**c**,**e**): tDCS group; (**b**,**d**,**f**): sham group.

HE staining (×200) revealed normal neuronal volume and morphology in both groups, with no evidence of degeneration, necrosis, or inflammatory cell infiltration. Notably, the sham group exhibited a higher neuronal density compared with the tDCS group, suggesting mild neuronal hyperplasia in the ACC of untreated EM rats. TEM analysis (×10,000 and ×30,000) demonstrated that sham-group neurons displayed mitochondrial swelling, cristae disruption, and partial chromatin condensation, whereas tDCS-group neurons showed relatively intact mitochondrial structures and preserved nuclear morphology, as shown in Figure 5. These observations suggest that tDCS exerts a protective effect on ACC neuronal ultrastructure in EM rats.

#### 3.3.3. Hippocampal CA1 Region

**Figure 6:**
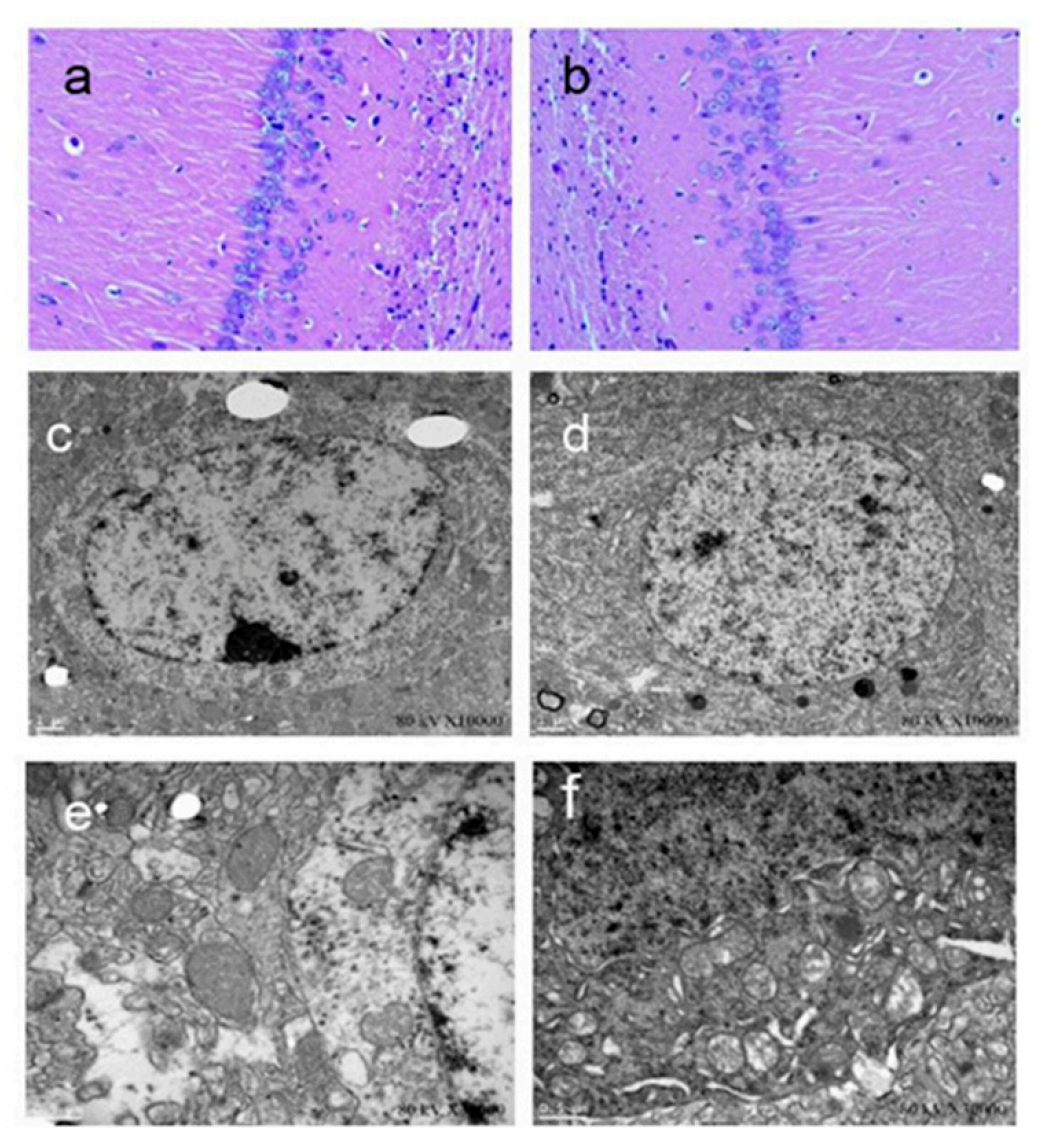
Morphological changes in the hippocampal CA1 region in EM rats (HE staining and TEM). HE staining (×200) and TEM ((**c**,**d**) ×10,000; (**e**,**f**) ×30,000). (**a**,**c**,**e**): tDCS group; (**b**,**d**,**f**): sham group.

HE staining (×200) showed that the volume and morphology of neurons in the hippocampal CA1 region were relatively normal in both groups, with no obvious pathological changes such as neuronal loss or degeneration. Consistent with the ACC findings, the neuronal density in the sham group was higher than that in the tDCS group. TEM analysis revealed that hippocampal neurons in the sham group exhibited mild mitochondrial swelling and cristae disorganization, while tDCS-group neurons displayed relatively well-preserved mitochondrial ultrastructure (in Figure 6), suggesting that tDCS may confer neuroprotective effects in the hippocampus.

#### 3.3.4. Thalamus

**Figure 7:**
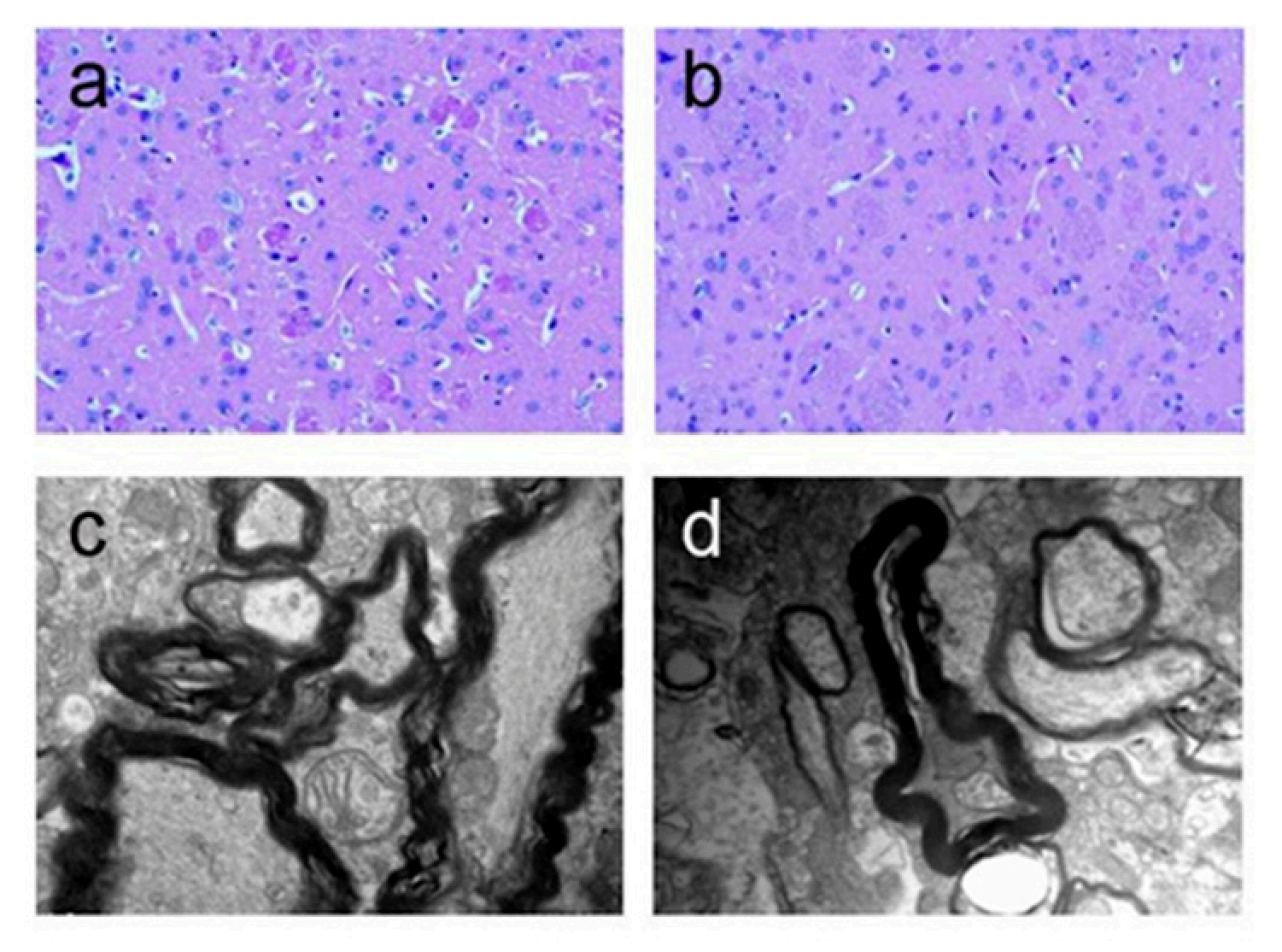
Morphological changes in the thalamus in EM rats (HE staining and TEM). HE staining (×200) and TEM (×30,000). (**a**,**c**): tDCS group; (**b**,**d**): sham group.

As shown in Figure 7, HE staining (×200) showed that the volume and morphology of thalamic neurons were relatively normal in both groups, with no obvious abnormalities such as inflammatory infiltration or neuronal degeneration. Neuronal density in the tDCS group was slightly higher than that in the sham group. TEM analysis revealed a striking difference in myelin sheath integrity: the sham group exhibited obvious myelin lamellae separation, loosening, and focal disruption, whereas the tDCS group demonstrated relatively intact myelin sheath architecture. These findings indicate that tDCS preserves thalamic myelin integrity, which may contribute to the maintenance of normal nociceptive signal transmission.

### 3.4. Effects of tDCS on Caspase-1 and IL-1β Expression in Ectopic Lesions

**Figure 8:**
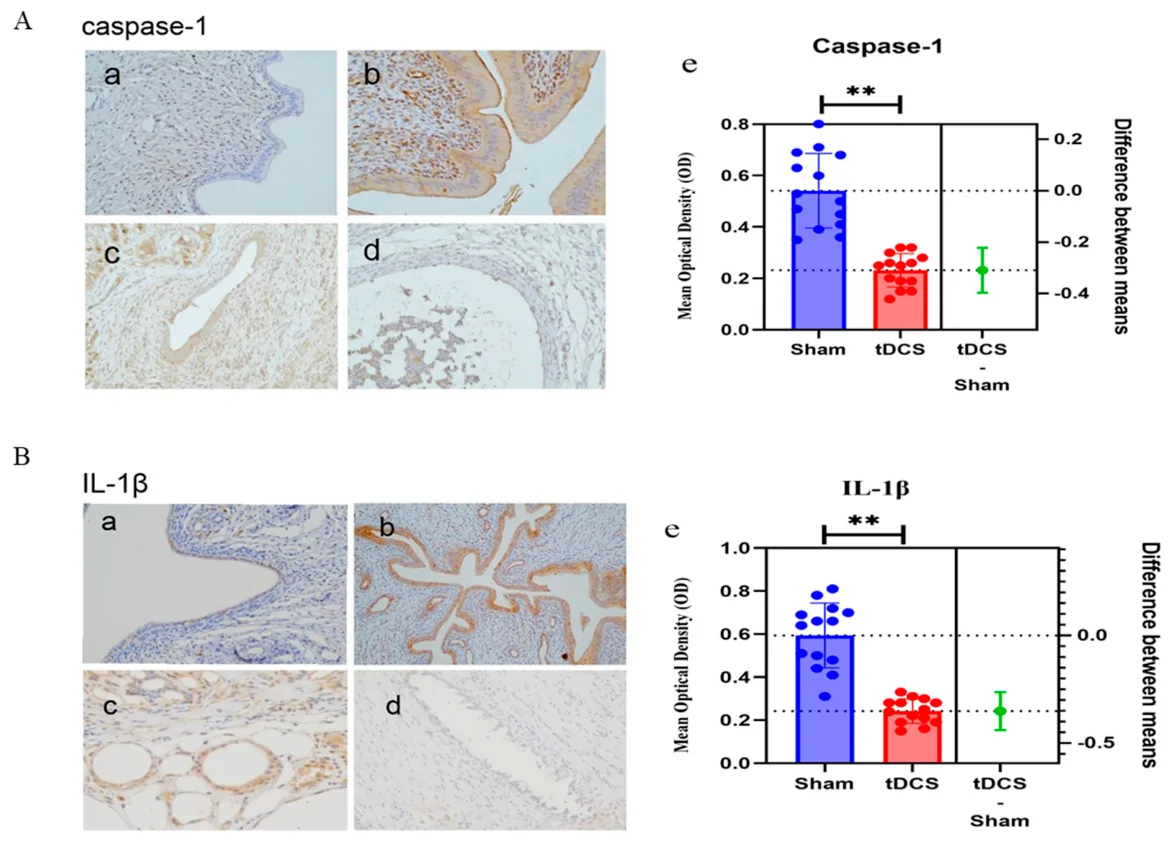
(**A**). Caspase−1 expression in ectopic lesions detected by IHC staining and quantitative analysis. (**a**) Normal endometrium (control), Caspase−1 negative; (**b**) endometriotic endometrium, Caspase−1 positive; (**c**) sham group ectopic cyst tissue, Caspase−1 positive; (**d**) tDCS group ectopic cyst tissue, Caspase−1 weakly positive (×200); (**e**) Caspase−1 AOD value (tDCS: 0.23 ± 0.03; sham: 0.54 ± 0.08). Data: $\bar{x}$ ± s (n = 14); ** *p* < 0.001 vs. sham (t = 7.285). (**B**). IL-1β expression in ectopic lesions detected by IHC staining and quantitative analysis. (**a**) Normal endometrium (control), IL-1β negative; (**b**) endometriotic endometrium, IL-1β positive; (**c**) sham group ectopic cyst tissue, IL-1β positive; (**d**) tDCS group ectopic cyst tissue, IL-1β weakly positive (×200); (**e**) IL-1β AOD value (tDCS: 0.24 ± 0.04; sham: 0.59 ± 0.09). Data: $\bar{x}$ ± s (n = 14); ** *p* < 0.001 vs. sham (t = 8.158).

As shown in Figure 8, Immunohistochemical (IHC) staining was employed to detect Caspase-1 and IL-1β expression in EM rat ectopic lesions, with normal endometrium from sham-operated rats serving as the negative control. Staining was evaluated under a light microscope (×200), with brownish-yellow granules in the cytoplasm or nucleus indicating positive expression. Quantitative analysis was performed using Image-Pro Plus 6.0 (Media Cybernetics, Rockville, MD, USA), with the average optical density (AOD) as the measurement indicator.

#### 3.4.1. Caspase-1

IHC staining revealed negative Caspase-1 expression in normal endometrium (no obvious positive staining). The sham group exhibited pronounced positive staining in the cytoplasm of ectopic glandular epithelial and stromal cells (high expression), whereas the tDCS group showed only weak positive staining (low expression). Quantitative analysis demonstrated that the AOD of Caspase-1 in the tDCS group (0.23 ± 0.03) was significantly lower than that in the sham group (0.54 ± 0.08) (t = 7.285, *p* < 0.001), indicating that tDCS effectively suppresses Caspase-1 expression in ectopic lesions (Figure 8A).

#### 3.4.2. IL-1β

IL-1β expression in ectopic lesions followed a pattern similar to that of Caspase-1: negative in normal endometrium (consistent with Caspase-1 staining), pronounced brownish-yellow positive staining (high expression) in the cytoplasm of ectopic cells in the sham group, and weak light-brown positive staining (low expression) in the tDCS group. Quantitative analysis showed that the AOD of IL-1β in the tDCS group (0.24 ± 0.04) was significantly lower than that in the sham group (0.59 ± 0.09) (t = 8.158, *p* < 0.001), confirming that tDCS effectively downregulates IL-1β expression in ectopic lesions (Figure 8B).

### 3.5. Effects of tDCS on Inflammatory Factors and Inflammasomes in Serum of EM Rats

**Figure 9:**
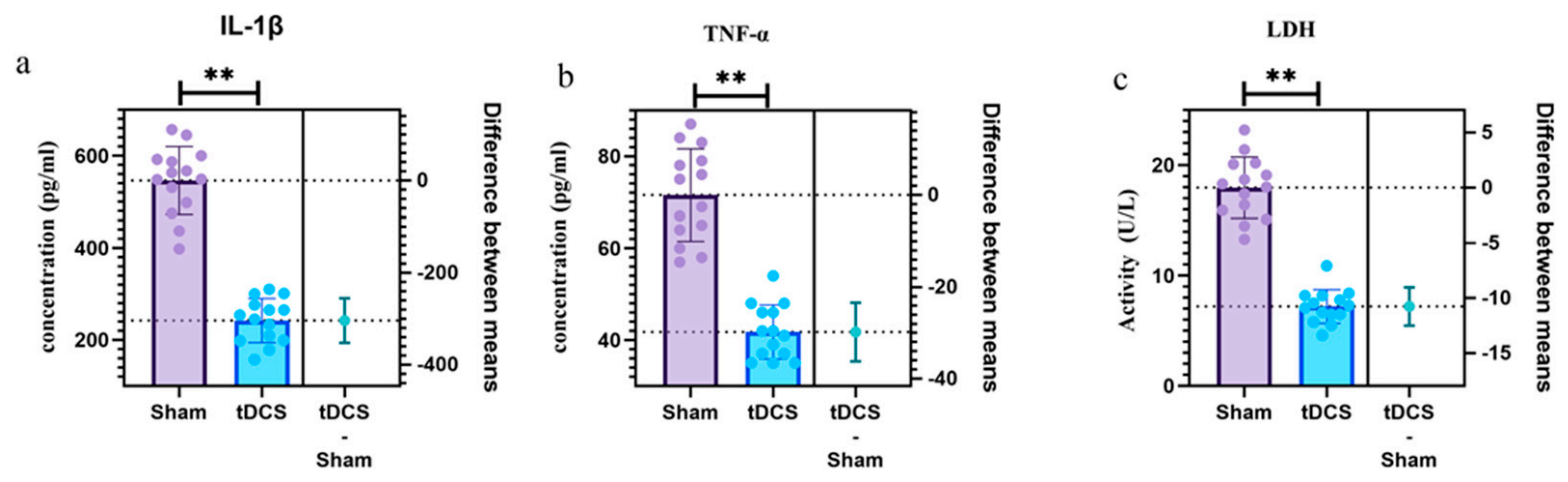
Quantitative comparison of serum IL−1β and TNF−α concentrations and LDH activity between the two groups. (**a**) Comparison of serum IL−1β concentration (t = 12.90, *p* < 0.001); (**b**) comparison of serum TNF−α concentration (t = 9.57, *p* < 0.001); (**c**) comparison of serum LDH activity (t = 12.72, *p* < 0.001). Data are expressed as $\bar{x}$ ± s (n = 14 per group); ** *p* < 0.001 vs. sham group. Statistical analysis: independent samples *t*-test; Software: GraphPad Prism 10.

Enzyme-linked immunosorbent assay (ELISA) was employed to measure the serum levels of inflammatory factors (IL-1β, TNF-α, and LDH) in EM model rats before and after tDCS treatment, to further elucidate the regulatory effect of tDCS on the systemic inflammatory response in EM. All assays were performed in strict accordance with the manufacturer’s instructions, and each sample was analyzed in duplicate (Figure 9).

## 4. Discussion

This study demonstrated that transcranial direct current stimulation (tDCS) exerts a significant analgesic effect on endometriosis (EM)-associated chronic pelvic pain by modulating the NLRP3 pyroptosis pathway through a novel brain–lesion axis regulatory mechanism. By integrating a randomized sham-controlled clinical trial with preclinical mechanistic investigations in an EM rat model, we provide convergent evidence that tDCS simultaneously suppresses peripheral inflammatory signaling and central neuroimmune remodeling. The clinical findings confirm that tDCS significantly reduces pain intensity and improves the quality of life in EM patients, while the animal experiments elucidate the underlying molecular mechanisms involving the NLRP3/Caspase-1/IL-1β pyroptotic cascade and its downstream effects on mitochondrial integrity, microglial activation, and myelin preservation in pain-related brain regions [23].

Our clinical results provide direct evidence that tDCS effectively alleviates endometriosis (EM)-related chronic pelvic pain and improves patients’ quality of life. Forty EM patients were randomized to active tDCS or sham stimulation, with well-balanced baseline characteristics (all *p* > 0.05) to minimize confounding bias. Following 10 days of intervention, the tDCS group exhibited a significant reduction in EPS scores from 7.25 ± 1.14 to 3.65 ± 0.67 (*p* < 0.001), while the EHP-30 scores decreased from 74.92 ± 6.78 to 39.12 ± 5.09 (*p* < 0.001). In contrast, the sham group showed no significant changes in either outcome measure. These findings are consistent with previous reports demonstrating the analgesic efficacy of tDCS in chronic pain conditions, including fibromyalgia and neuropathic pain. Importantly, the analgesic effect of tDCS was sustained at 10 days post-treatment, suggesting a durable modulation of pain pathways rather than a transient placebo response.

Animal experiments complemented and extended the clinical findings by elucidating the peripheral regulatory mechanism by which tDCS inhibits the NLRP3 pyroptosis pathway in EM. The EM rat model was successfully established via autologous uterine tissue transplantation, with model validity confirmed by ectopic lesion formation and significant hyperalgesia (reduced hot plate latency). tDCS treatment significantly prolonged the withdrawal latency (from 11.32 ± 0.78 s to 27.07 ± 1.66 s, *p* < 0.001), demonstrating a robust analgesic effect. Immunohistochemical analysis revealed that tDCS significantly downregulated Caspase-1 (AOD: 0.23 ± 0.03 vs. 0.54 ± 0.08, *p* < 0.001) and IL-1β (AOD: 0.24 ± 0.04 vs. 0.59 ± 0.09, *p* < 0.001) expression in ectopic lesions, indicating suppression of the NLRP3/Caspase-1/IL-1β pyroptotic cascade. Serum ELISA further confirmed significant reductions in the IL-1β, TNF-α, and LDH levels, reflecting attenuated systemic inflammation and reduced pyroptotic cell death.

Morphological observations of pain-related brain regions in EM rats revealed the central regulatory mechanism by which tDCS protects neural structure and function and inhibits central neuroinflammation, representing another key link in the brain–lesion axis [24]. The transmission electron microscopy (TEM) results demonstrated that tDCS preserved mitochondrial ultrastructure in ACC neurons, mitigated microglial activation, and maintained myelin sheath integrity in the thalamus. These findings suggest that tDCS exerts a neuroprotective effect on central pain-processing circuits, potentially by attenuating the neuroinflammatory cascade downstream of NLRP3 activation. The observed protection of thalamic myelin is of particular significance, as demyelination in sensory relay nuclei can amplify nociceptive signal transmission and contribute to central sensitization.

The most important novel finding of this study is the proposal of the brain–lesion axis regulatory model, which elucidates the bidirectional modulation mechanism of tDCS on EM pain by targeting the NLRP3 pyroptosis pathway. tDCS exerts a top-down regulatory effect on peripheral ectopic lesions through the activation of descending pain inhibitory pathways (DLPFC–PAG–RVM–spinal dorsal horn), while peripheral inflammatory signals transmit bottom-up to central pain networks via visceral afferent nerves and circulating cytokines crossing the blood–brain barrier. This bidirectional communication forms a self-reinforcing “pain–inflammation” cycle that tDCS disrupts by simultaneously suppressing peripheral pyroptosis and central neuroinflammation [25]. The cholinergic anti-inflammatory pathway (CAP), mediated by the vagus nerve, and the hypothalamic–pituitary–adrenal (HPA) axis may serve as key intermediate conduits for this brain–lesion communication [26,27]. It should be emphasized, however, that the present findings demonstrate temporal synchronicity between peripheral and central inflammatory changes following tDCS, rather than definitive causal relationships. The bidirectional regulatory loop remains a testable hypothesis warranting targeted interventional studies.

This descending inhibitory circuit is well established in the neurophysiological literature: activation of the PAG recruits endogenous opioid and serotonergic systems that govern RVM ON/OFF-cell activity to inhibit spinal nociceptive transmission [28]. In vivo human neuroimaging further supports engagement of this descending system by tDCS: combining placebo and active tDCS recruits the endogenous μ-opioid system within the PAG, as demonstrated by carfentanil positron-emission tomography [29]; in a rodent neuropathic-pain model, tDCS reduced microglial activation and promoted a shift toward the anti-inflammatory M2 phenotype, consistent with the attenuation of central neuroinflammation [30].

Compared with conventional EM pain treatments, tDCS offers unique advantages in targeting the neuroimmune core of EM pain. Nonsteroidal anti-inflammatory drugs (NSAIDs) provide only transient symptomatic relief and carry a substantial risk of gastrointestinal damage with long-term use [31]; gonadotropin-releasing hormone agonists (GnRH-a) suppress lesion growth but are associated with significant adverse effects and high recurrence rates [32]; and surgical excision, while effective for lesion removal, carries recurrence rates of 30–50% and risks compromising ovarian reserves [33,34]. In contrast, tDCS is noninvasive, well-tolerated, and directly targets the neuroimmune mechanisms driving chronic pain, offering a fundamentally different therapeutic approach. Furthermore, tDCS can be combined with pharmacological or surgical treatments as part of a multimodal pain management strategy [35].

**Figure 10:**
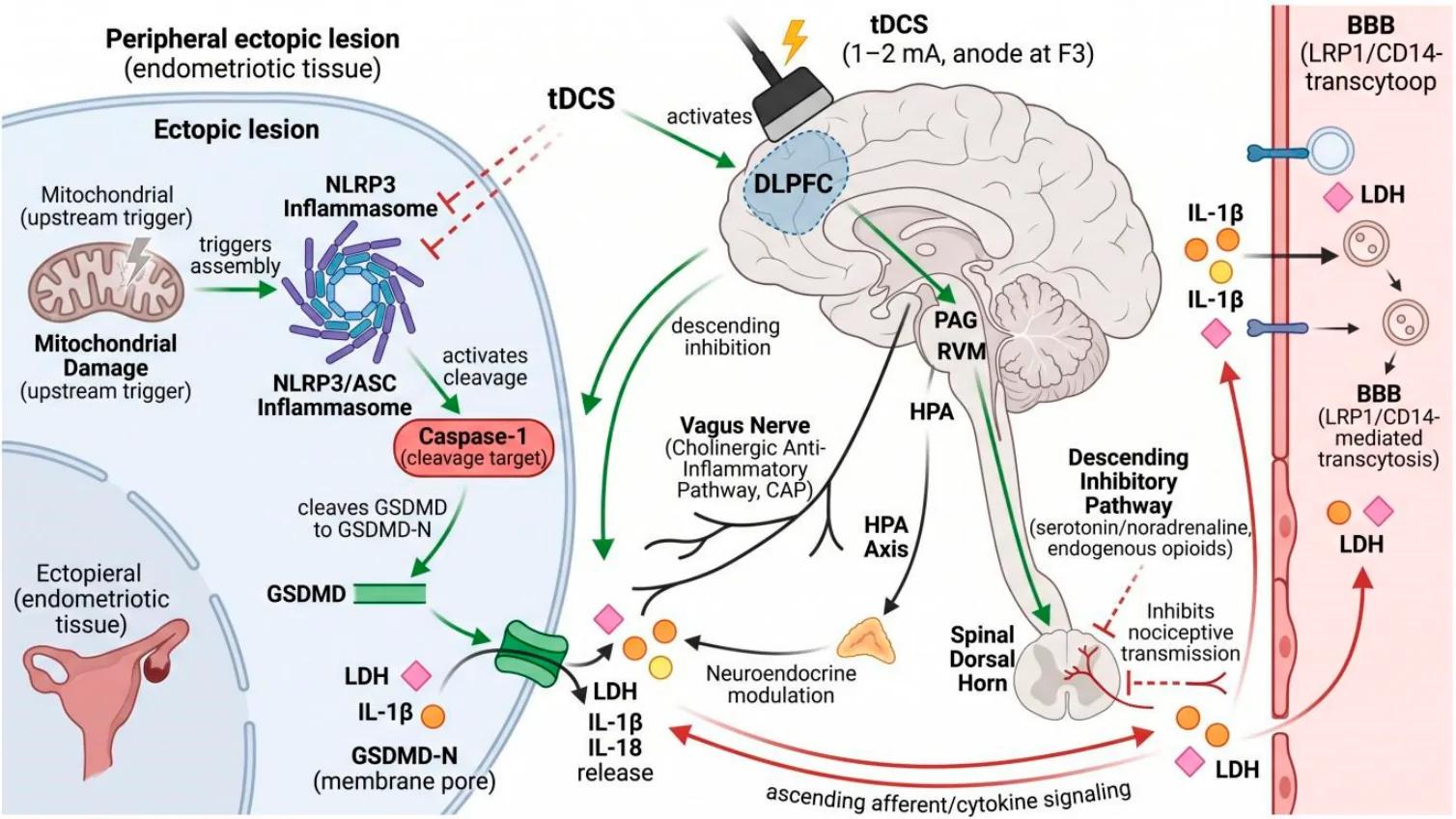
Schematic diagram of the scientific hypothesis for tDCS in treating endometriosis (EM)-related pain. Central to the model is the NLRP3 inflammasome–Caspase-1-mediated pyroptotic cascade in ectopic lesions and pain-related brain regions: NLRP3/ASC inflammasome assembly activates Caspase-1, which cleaves GSDMD to its N-terminal fragment (GSDMD-N), forms plasma-membrane pores, and drives LDH release together with maturation and secretion of IL-1β and IL-18; mitochondrial damage serves as an upstream trigger. tDCS (1–2 mA, anode at F3) inhibits NLRP3 activation, thereby reducing Caspase-1 cleavage, GSDMD-N pore formation, LDH leakage, and IL-1β/IL-18 release, alleviating both peripheral ectopic-lesion inflammation and central neuroimmune remodeling. This peripheral pyroptotic signal and the central analgesic response are linked through a closed-loop brain–lesion neuroimmune axis: (i) top-down, tDCS activates the DLPFC→PAG→RVM→spinal dorsal horn descending inhibitory pathway (serotonin/noradrenaline, endogenous opioids) that suppresses nociceptive transmission; (ii) the vagus nerve cholinergic anti-inflammatory pathway (CAP) and the hypothalamic–pituitary–adrenal (HPA) axis act as intermediate conduits between cortex and periphery; (iii) bottom-up, visceral afferents and circulating cytokines (e.g., IL-1β, LDH) cross the blood–brain barrier (LRP1/CD14-mediated transcytosis) to reach central pain networks. The bidirectional loop is proposed as a testable hypothesis warranting targeted interventional studies.

This study also provides potential objective biomarkers for the clinical application and efficacy evaluation of tDCS in EM pain treatment. Importantly, this study is the first to anchor the mechanism of action of tDCS to the NLRP3 inflammasome pathway: tDCS delivers weak current via a specific electrode placement protocol to inhibit NLRP3 activation, reduce downstream IL-1β and IL-18 release and LDH elevation, alleviate inflammation and pyroptosis, and thereby relieve EM-related pain. The degree of pain relief correlates positively with the reduction in these inflammatory factors, supporting the NLRP3 pathway as a core analgesic mechanism of tDCS in EM. These findings suggest that serum NLRP3, Caspase-1, IL-1β, and LDH levels may serve as candidate biomarkers for predicting treatment response and guiding the individualized adjustment of tDCS stimulation protocols in clinical practice, significantly contributing to the standardized and personalized application of tDCS in EM pain treatment (Figure 10).

In conclusion, this study not only confirms that tDCS alleviates endometriosis (EM) pain by bidirectionally inhibiting the NLRP3 pathway through the brain–lesion axis but also establishes a theoretical bridge between neuromodulation and immune regulation. Despite challenges in individualized treatment protocols and long-term management, the noninvasive nature and favorable side-effect profile of tDCS position it as a key driving force for a paradigm shift in the treatment of EM-associated pain.

## 5. Conclusions

Despite the important findings, this study has certain limitations. First, the sample size was determined by a priori power analysis for the primary outcome; however, the single-center exploratory design with a moderate sample size may limit the generalizability of the results. Second, the study lacks long-term follow-up data, and the duration of the analgesic effect of tDCS, as well as its long-term impact on the neuroimmune function of EM patients and rats, requires further investigation. Third, the study employed a single tDCS stimulation protocol, and the optimal stimulation parameters (e.g., current intensity, stimulation duration, frequency, and electrode placement) for EM pain have not been systematically evaluated. Fourth, the bidirectional brain–lesion regulatory axis is supported by correlational evidence rather than definitive causal relationships established through targeted blocking assays. Fifth, the pyroptosis detection panel was incomplete, measuring downstream Caspase-1 and IL-1β but not upstream NLRP3/ASC assembly or terminal GSDMD-N cleavage. Sixth, the clinical study relied on subjective self-reported questionnaires without circulating pyroptosis biomarkers. Seventh, the DLPFC–PAG–RVM descending analgesic circuit was not directly verified by experimental evidence. Future studies should expand the clinical sample size, conduct long-term follow-up to evaluate the sustained efficacy and safety of tDCS, explore optimal stimulation protocols through gradient parameter experiments, and perform targeted interventional studies (e.g., vagotomy, local NLRP3 inhibition, PAG/RVM tissue collection) to establish definitive causal relationships within the brain–lesion neuroimmune axis. In addition, the clinical cohort was diagnosed by imaging (transvaginal ultrasound and MRI) without systematic laparoscopic r-AFS staging or histopathological lesion-subtype classification, as most participants had not undergone surgical intervention; consequently, anatomical staging, lesion-subtype stratification, and covariate-adjusted regression incorporating these variables could not be performed.

## Figures and Tables

**Table 1:** Comparison of baseline characteristics, EPS and EHP-30 scores between the two groups at different time points ($\bar{x}$ ± s, n = 40).

**Group** **n = 40**	**VAS** **Score**	**Age (Years)**	**t Value**	***p* Value**	
tDCS n = 20	8 ± 1.6	37 ± 2.1	0.42 (VAS); 1.58 (Age)	0.68 (VAS); 0.12 (Age)	
Sham n = 20	8 ± 1.4	38 ± 2.3			
	**EPS** **Score**				
	Baseline	Day 1	Day 10	t value	*p* value
tDCS n = 20	7.25 ± 1.14	4.75 ± 0.85	3.65 ± 0.67	9.82	<0.001
Sham n = 20	7.30 ± 1.21	6.85 ± 1.32	7.05 ± 1.28		
	**EHP-30** **Score**				
	Baseline	Day 1	Day 10	t value	*p* value
tDCS n = 20	74.92 ± 6.78	51.85 ± 5.87	39.12 ± 5.09	10.56	<0.001
Sham n = 20	74.56 ± 6.69	72.98 ± 6.51	72.65 ± 6.40		

Note: VAS = Visual Analog Scale; t values and *p* values were calculated by independent samples *t*-test; *p* > 0.05 indicates no significant difference between the two groups at baseline. *p* < 0.05 compared with the same group at baseline; *p* < 0.05 compared with the same group 1 day after treatment; t value and *p* value were calculated by independent samples *t*-test between the two groups at the last follow-up (10 days after treatment); EPS = Endometriosis Pain Scale. EHP-30 = Endometriosis Health Profile-30.

## Data Availability

The datasets used and/or analyzed during the current study are available from the corresponding author on reasonable request.
